# Multi-omics time-series analysis in microbiome research: a systematic review

**DOI:** 10.1093/bib/bbaf502

**Published:** 2025-10-06

**Authors:** Moiz Khan Sherwani, Matti O Ruuskanen, Dylan Feldner-Busztin, Panos Nisantzis Firbas, Gergely Boza, Ágnes Móréh, Tuomas Borman, Pande Putu Erawijantari, István Scheuring, Shyam Gopalakrishnan, Leo Lahti

**Affiliations:** Center for Evolutionary Hologenomics, GLOBE Institute, University of Copenhagen, Øster Farimagsgade 5, 1353 Copenhagen K, Denmark; Department of Computing, University of Turku, 20014, Turku, Finland; Champalimaud Research, Champalimaud Centre for the Unknown, Av. Brasília, 1400-038 Lisbon, Portugal; Champalimaud Research, Champalimaud Centre for the Unknown, Av. Brasília, 1400-038 Lisbon, Portugal; HUN-REN, Institute of Evolution, Centre for Ecological Research, H-1113 Budapest, Karolina Road 29, Hungary; HUN-REN, Institute of Evolution, Centre for Ecological Research, H-1113 Budapest, Karolina Road 29, Hungary; Department of Computing, University of Turku, 20014, Turku, Finland; Department of Computing, University of Turku, 20014, Turku, Finland; HUN-REN, Institute of Evolution, Centre for Ecological Research, H-1113 Budapest, Karolina Road 29, Hungary; Center for Evolutionary Hologenomics, GLOBE Institute, University of Copenhagen, Øster Farimagsgade 5, 1353 Copenhagen K, Denmark; Department of Computing, University of Turku, 20014, Turku, Finland

**Keywords:** time-series, multi-omics, host-associated microbiomes, statistical modeling, machine learning

## Abstract

Recent developments in data generation have opened up unprecedented insights into living systems. It has been recognized that integrating and characterizing temporal variation simultaneously across multiple scales, from specific molecular interactions to entire ecosystems, is crucial for uncovering biological mechanisms and understanding the emergence of complex phenotypes. With the increasing number of studies incorporating multi-omics data sampled over time, it has become clear that integrated approaches are pivotal for these efforts. However, standard data analytical practices in longitudinal multi-omics are still shaping up and many of the available methods have not yet been widely evaluated and adopted. To address this gap, we performed the first systematic literature review that comprehensively categorizes, compares, and evaluates computational methods for longitudinal multi-omics integration, with a particular emphasis on four categories of the studies: (i) host and host-associated microbiome studies, (ii) microbiome-free host studies, (iii) host-free microbiome studies, and (iv) methodological framework studies. Our review highlights current methodological trends, identifies widely used and high-performing frameworks, and assesses each method across performance, interpretability, and ease of use. We further organize these methods into thematic groups—such as statistical modeling, machine learning, dimensionality reduction, and latent factor approaches—to provide a clear roadmap for future research and application. This work offers a critical foundation for advancing integrative longitudinal data science and supporting reproducible, scalable analysis in this rapidly evolving field.

## Introduction

Multicellular organisms coexist with microbes, collectively constituting a *holobiont* [1]. For a holistic understanding of the host organism, we need to understand the network of interactions between the host and its microbiomes. Some of the most important questions related to multicellular hosts are e.g. how they maintain their homeostasis, react to changing environments, defend against infections, and how the interactions between the hosts and their microbiomes contribute to these processes. To answer these questions, we should analyze the host genome (complete DNA sequence), epigenome (chemical modifications to DNA), transcriptome (all RNA transcripts), proteome (set of proteins expressed in an organism), metabolome (complete set of metabolites), and other aspects of the system in parallel. Thus, the collection of multi-omics data from the host and host-associated microbiomes and the development of multi-omics analysis techniques have emerged as an active research topic [2]. Despite the progress, revealing causal relations and accounting for temporal variation in multi-omics studies necessitate sampling across different time points and treatment conditions.

Multi-omics data and the related analysis methods are heterogeneous. The various ’omics represent very different types of biological molecules. *Meta-genomics* involves the comprehensive sequencing of all microbial genomes within a sample, enabling the reconstruction of functional potential and the community structure of the microbiome [3]. *Transcriptomics* measures RNA transcripts to estimate the relative expression of genes [4]. *Proteomics* includes quantitative measures of the different proteins [5], while *meta-taxonomics* aims to characterize all microbial taxa in a sample [6], often through 16S rRNA gene amplicon sequencing [7]. *Genomics* enables us to determine whether mutations are present at specific positions in the genome and *epigenomics* informs us on differences in gene regulation [8]. Each of these methods has its own technical challenges and resulting biases, e.g. the identification and quantification of proteins with low abundance in *proteomics* [5], selecting an appropriate preprocessing method in *transcriptomics* [9], and deciding how to define the units of analysis in *meta-taxonomics* [10]. There are also further issues of high dimensionality (large number of features or variables compared with the relatively small number of samples), high stochasticity or noise (random variations that obscure true signal), and batch effects (systematic variations introduced by differences in experimental conditions). Taken together, these and other properties of the data can make the interpretation and use of the data difficult [11]. Furthermore, matching the samples and features between the complementary ’omics is necessary for joint analysis but not always straightforward. A description of these challenges has been given by Chalise *et al*. [12].

**Figure 1:**
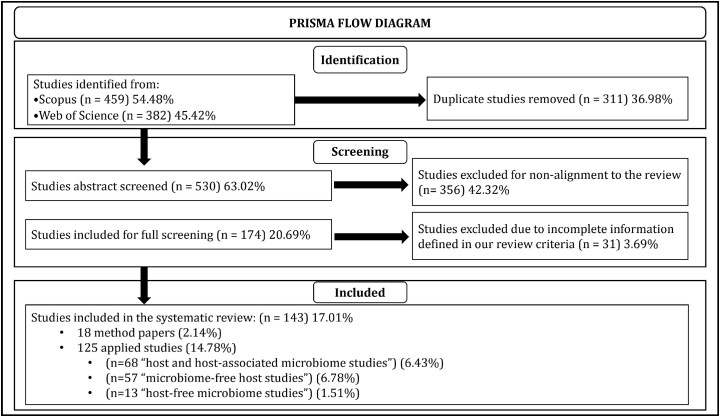
PRISMA flow diagram illustrating the study selection process for this review.

Including the temporal dimension brings in an additional layer of challenges in terms of data collection and analysis. It might be impossible to comprehensively analyze the same entity, such as a developing organism, at different time points. In such cases, it might be necessary to perform a pseudo-time series, i.e. a time-series with different samples from a (relatively) homogeneous population sampled at different time points. Additionally, temporal multi-omics can provide various benefits, such as balancing out individual variability [13] and provide a dynamic view on the holobiont.

Thus, to comprehend the multitude of interactions occurring within the holobiont over time, it is necessary to employ temporal version of multi-omics analysis techniques. This review provides a systematic overview of data analytical methods in longitudinal multi-omics and highlights emerging topics for future research.

In addition to microbiome research, longitudinal multi-omics analysis has become a powerful method in wider biological applications. In personalized medicine, monitoring molecular alterations over time facilitates early illness diagnosis, individualized treatment planning, and ongoing therapy assessment [14]. Applications in cancer, neurological diseases, and metabolic disorders have illustrated the efficacy of time-resolved omics profiling in revealing causative processes and treatment-responsive biomarkers. These advancements underscore the extensive significance and versatility of time-series multi-omics frameworks.

## Systematic review method

### Preliminary systematic search and screening of studies

**Figure 2:**
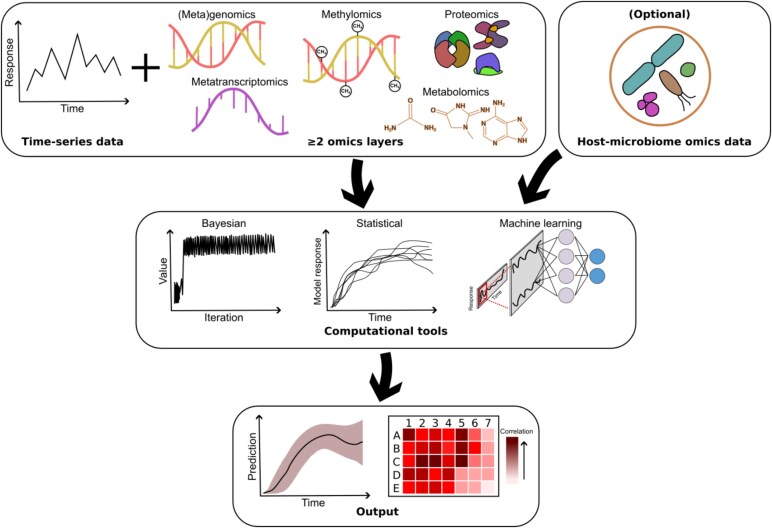
Overview of the study design in this systematic review: Omics layers (e.g., transcriptomics, genomics, meta-genomics) are integrated as multi-omics time-series data (minimum two layers) with optional host-associated microbiome data, and analyzed through statistical, ML, and DL methods to generate outputs such as predictions and correlations, with the diagram outlining the full process from data collection to computational processing and final output generation within the reviewed studies.

We followed the PRISMA guidelines [15] (Fig. 1) to ensure transparency and reproducibility, given their broad acceptance for systematic reviews in biomedical research. Figure 2 shows specific inclusion/exclusion criteria. We defined multi-omics data as a combination of two or more ’omics datasets that included longitudinal measurements as real or pseudo time-series. It was vital to include a research that includes pseudo-time series since sampling can sometimes be destructive, which makes it hard to collect full longitudinal observations [16]. The search for relevant literature was conducted on 15 June 2024, using the key “multi-omics (“time series” OR “over time” OR “temporal” OR “longitudinal”)” in all domains of the Web of Science. The Scopus database was queried using the search term “TITLE-ABS-KEY [“multi” AND “omics” AND (“time series” OR “across time” OR “temporal” OR “longitudinal”)].” We restricted the searches to original studies written in English and excluded review studies. This yielded 382 entries from Web of Science and 459 entries from Scopus. After manually identifying and eliminating 311 duplicates, 530 distinct records remained for analysis. Based on abstract screening, we excluded studies that did not align within the defined scope. The remaining 174 studies underwent full-text screening, during which we excluded further 31 studies out of 174 studies due to incomplete information as defined in our study design Fig. 2. A total of 143 studies fulfilled the criteria established for this review.

### Evaluation of studies

The 143 studies that met our systematic review criteria consisted of 125 (87%) applied studies and 18 (13%) methodological studies. Among the 125 applied studies, 55 included “host and host-associated microbiome data”—these studies investigate both the host and its associated microbial communities (Table 1). Of the remaining 70 studies, 57 included “microbiome-free host data”—these studies focus exclusively on the host, analyzing host *genomics*, *transcriptomics*, *proteomics*, or *meta-bolomics* without considering microbial data (Table 2). Finally, there were 13 studies that focused on “host-free microbiome data”—these studies examine microbial communities in environments or contexts where a host is not involved, such as free-living or environmental microbiomes (Table 3). Each study was evaluated by at least two authors to ensure consistency. For the applied studies, we systematically summarized key aspects, including the types of samples analyzed, the frequency and duration of sampling, the types of ’omics data used, and the analytical approaches employed.

For the methods-based studies, we performed a qualitative assessment based on established criteria: predictive performance, interpretability, and ease of installation/use. These criteria were selected to address fundamental considerations for the actual implementation and usability of methods in multi-omics research. Predictive performance was emphasized to guarantee the reliability and accuracy of results, while interpretability evaluated the capacity of each approach to produce understandable and significant insights for users. User-friendly implementation and the ease of installation are essential considerations for the accessibility of a wider scientific audience. Furthermore, we evaluated the development and maintenance activities of each technique in order to further assess the robustness and long-term availability. This assessment included data from the primary studies and associated resources, including code repositories, tutorials, and online documentation (Table 4). Collectively, these factors underscore the necessity of choosing methods that are robust, pragmatic, and consistently maintained.

**Table 1:** Overview of multi-omics “host and host-associated microbiome” studies, including authors, year of study, sample type, temporal sampling frequency, data types (Genomics, Transcriptomics, Proteomics, Meta-bolomics, Meta-genomics, Meta-taxonomics and Others), and applied modeling or ML approaches

Authors (Year)	Number of samples	Sample type	Time-series (frequency)	G	T	P	MB	MG	MT	Other	Modeling approach	ML
Thaiss *et al*. [17] 2016	-	blood, mucosa, serum	days (hourly)	-	x	-	x	-	-	Epigenome	JTK_cycle, KW, WT	-
Skarke *et al*. [18] 2017	60	blood, saliva, rectal swab, plasma, serum	4 months (alternate weeks)	-	x	x	x	x	-	-	MCPT, PCA, VCA, circadian multiresolution analyses, cosinor method, IPA	x
Piening *et al*. [13] 2018	23	blood, feces	90 - 180 days (90 timepoints)	x	x	x	x	x	-	-	RF, AB, LASSO, ENet, 10CV, PE, FCC, ANOVA, T-test	x
Zhou *et al*. [19] 2019	106	blood, nasal, feces	4 years (tri-monthly for baselines, weekly)	x	x	x	x	x	x	-	LMM, MLR, LR, SVM	x
Poyet *et al*. [20] 2019	3632 WGS, 80 multi-omics	feces	18 months (daily)	-	-	-	-	x	x	-	PR, LMM, PERMANOVA, PCA, DS	x
Rechenberger *et al*. [21] 2019	56	feces	months (weekly)	-	-	-	-	x	-	x	Jaccard similairty, PC	-
Lloyd-Price *et al*. [22] 2019	six host, 24 microbiome	blood, intestine, feces, biopsy	months (weekly to monthly)	x	x	-	-	x	-	-	PCA, MT, PERMANOVA, BCD, LMM	x
Paix *et al*. [23] 2019	three	surface of the thalli	6 months (monthly)	-	-	-	x	-	x	-	PCA,sPLS-DA, PERMANOVA, ANOVA, BCD, PCoA	x
Gierse *et al*. [24] 2020	three	Feces, ileum, proximal colon, distal colon	30 days	-	-	x	x	-	x	-	NMDS, DS	x
Hu *et al*. [25] 2020	76	blood, renal tissues, feces, serum	28 days (daily)	-	-	-	x	-	x	-	PCA, LOWESS, OPLS-DA, LC-MS, T-test, WT, KW, PC	x
Contrepois *et al*. [26] 2020	36	blood, plasma, feces	1 h (minutely)	-	x	x	x	-	x	Lipidome	LM, FCC, LR, SVM, RR	x
Shannon *et al*. [27] 2020	15	blood, feces	7 months (daily to monthly)	-	x	x	-	x	-	Epigenome	PCA, DIABLO	x
Ta *et al*. [28] 2020	63	feces	12 months (week3 & every 3 months)	-	-	-	x	x	-	Meta-trascriptomics	LMM, PCA	x
Taylor *et al*. [29] 2020	115 longitudinal study, 8000 one time study	feces	4 weeks (weekly)	-	-	-	x	x	-	-	PLS-DA, SFPCA, AD, BD, WT, mmvec, Songbird	x
Metwaly *et al*. [30] 2020	20	feces	5 years (monthly to yearly)	-	-	-	-	x	x	-	LDA, RF, PCA, Volcano plots, T-test, ANOVA	x
Mars *et al*. [31] 2020	77	blood, colonic mucosal biopsy, feces, serum	months (monthly)	-	x	-	x	x	-	Epigenome	PCoA, MCMA (Maaslin), Lasso	x
Leonard *et al*. [32] 2020	31	blood, feces	6 months (monthly)	x	-	-	-	x	-	-	MAASLIN, SC	x
Gierse *et al*. [33] 2021	six	feces, mucus	25 days (daily to weekly)	-	-	-	x	-	x	-	ANOVA, MDS, WT	x
Kim *et al*. [34] 2021	57	blood, feces, serum	weeks, months (daily to monthly)	-	-	-	x	-	x	-	PCA, ST, LDA, MVC, LEfSE, BC-UPGMA, PP, GC–TOF–MS	x
Zimmer *et al*. [35] 2021	3558	blood, feces	years (monthly)	-	-	x	x	x	-	-	t-SNE, MOFA, PCA, T-test	x
Monaghan *et al*. [36] 2021	four	blood, feces, serum	1.5 months (weekly)	-	-	x	x	-	x	Epigenome	T-tests, KM, HC, SC	x
Laursen *et al*. [37] 2021	25 longitudinal study, 59 testing	feces	6 months (2-4 weeks)	-	-	-	x	x	-	-	PCA, AT, MT, CA, ANOVA, LMM	x
He *et al*. [38] 2021	13	feces, blood, tissue	94 days (daily-monthly)	-	-	x	-	-	x	-	PCA, HCA, WT, PC, MU	x
Conta *et al*. [39] 2021	one	Breastmilk, infant feces	4 - 10 month (day 103-175 breastmilk, day 219–268 feces)	-	-	-	x	x	-	-	PCA, sPLS-DA, multi-block PLS-DA	x
Sillner *et al*. [40] 2021	seven	feces	2 years (monthly)	-	-	-	x	x	-	-	sPLS-DA, PCA, SC, KW, WT	x
Revilla *et al*. [41] 2021	45 508	intestine, mucosa, biopsy	years (monthly)	x	x	-	-	x	-	-	Sparse regularized generalized canonical correlation analysis	x
Mihindukulasuriya *et al*. [42] 2021	50	feces	months (monthly)	-	x	-	-	x	-	-	CA, PCA, KW, LASSO	x
Monteleone *et al*. [43] 2021	40	feces	20 weeks (monthly)	-	-	-	x	-	x	-	ANOVA, PERMANOVA, Welch T-test, SC, LEFSE, KW, WT, LDA, HC, GA	x
Chen *et al*. [44] 2021	338	blood, feces, plasma	4 years	-	-	-	x	x	-	-	WT, PC, HC, LMM	x
Huang *et al*. [45] 2021	40	saliva	28 days (daily to weekly)	-	-	-	x	x	-	Immunomics	PCA, PERMANOVA, RF, WT, MCPT, SC, CN	x
Paix *et al*. [46] 2021	15	surface of the thalli	6 months (monthly)	-	-	-	x	-	x	-	FROGS workflow, sPLS-DA, DIABLO for CA, ANOVA, NMDS, PCA, PERMANOVA	x
Xiao *et al*. [47] 2022	24 juvenile, 16 adult, 230 giant pandas	juvenile: blood and intestinal, Adult: feces	1 time from different groups (days to years)	x	x	-	x	x	-	-	SI, PCoA, Ma, LE, T, TD, Tn, DESeq, KEGG, GOE, Mh, KEGG, eggNOG, NMDS, PCoA, BCD, PH, MetAn, ROC, MT, MO, sPLS-DA, Cy, PER, WT, MT	x
Cantoni *et al*. [48] 2022	49 (24 RRMS, 25 HC)	blood, feces	6 months (daily-monthly)	-	-	-	x	x	-	-	UPLC-MS, PCA, PERMANOVA, DESeq2, WT, Welch’s t-test, MT, RF, ENL, SVM	x
Dang *et al*. [49] 2022	70	blood, feces	9 months (monthly)	-	-	-	x	x	-	-	SC, sPLS-DA	x
Worby *et al*. [50] 2022	367 (with UTI: 197, controls: 170), urine samples: $n$=18	urine, blood, rectal swabs, feces	1 year (monthly)	-	x	-	-	x	-	-	LMM, BCD	x
Watzenboeck *et al*. [51] 2022	78	bronchoalveolar lavage	months (monthly)	-	-	-	x	x	-	-	dbRDA, PCoA, PCA,LMM, MAASLIN, RR	x
Baccarelli *et al*. [52] 2023	multiple samples	blood, saliva, urine, stool, tissues, biospecimens	-	x	x	x	x	-	-	epigenomics, exposomics	ML, statistical approaches and integration of multi-omics data	x
Liu *et al*. [53] 2023	44 captive giant pandas	Feces	cross-sectional study	-	-	-	-	-	-	-	Mfuzz clustering, KW, CA	-
Zoelzer *et al*. [54] 2023	95 (inc. five wildebeests + six tigers)	Feces	8 days	x	-	-	-	-	-	-	HC, LASSO, ANOVA, ANOSIM	x
Ambikaan *et al*. [55] 2023	30 (CCHFV), 22 HC	blood	3 time points	-	x	x	-	-	-	-	maSigPro, KEGG, Gaussian models	-
Symul *et al*. [56] 2023	30 + 200 nonpregnant, 39 + 96 pregnant	vaginal swabs	10 weeks (Nonpregnant (daily)), month 4 onwards (Pregnant (daily))	x	-	-	x	-	-	-	LDA, DADA2, LMM, LR, MCPT	x
Zhang *et al*. [57] 2023	45 with probiotics, 45 controls	Feces, blood	Baseline, 6 weeks	-	-	-	x	-	-	CBC, lymphocytes, cytokines	WT, UMAP, PERMANOVA, DA	x
Hornburg *et al*. [58] 2023	112, 1500 plasma	Blood, plasma	9 years (quarter yearly)	-	-	-	x	-	-	Lipidomics, Cytokines	KM, t-SNE, KNN based imputation, WGCNA, LMMs, GAMM	x
Osterdahl *et al*. [59] 2023	2561	Feces, swab	multiple timepoints	-	-	-	x	x	-	-	LR, WT, PERMANOVA, LMM, SC, HC	-
Watson *et al*. [60] 2023	109	Feces	multiple timepoints	x	-	-	-	x	-	Phylo-genomics	EA, LMM, Rao test statistics, uncorrected $P$-values, corrected q-values.	-
Attia *et al*. [61] 2023	48 male Sprague– Dawley rats	Feces, colonic tissue	1 month (weekly)	x	-	-	x	x	-	-	T-test, ANOVA, WT, KW, SC, PERMANOVA, Dunn test	-
Gates *et al*. [62] 2023	10 (five Balb/c and five C57BL/6)	Feces	17 weeks	-	-	x	-	x	x	-	BCD, PCoA, ANOSIM, SC	-
Thormar *et al*. [63] 2024	44 zebrafish(four albino, 20 mosaic, and 20 wild-type)	Feces	-	-	-	-	x	-	x	Holo-genomics	CRISPR, DADA2, Decontam, LULU, Metacoder, PCA, PERMANOVA, KW, WT, GLM	-
Luo *et al*. [64] 2024	20 dairy cows(10 healthy + 10 hyperketonemic)	Fecal, blood	multiple timepoints pre/post calving	-	-	-	x	x	x	-	time-series analysis, WT, T-test, SC, PCoA, RF, ROC, MT, Adonis analysis	x
Schaan *et al*. [65] 2024	48	Feces	two distinct time points	x	-	-	-	x	x	-	Kraken2, InStrain, Ancom, CA, Alpha/Beta diversity	-
Laue *et al*. [66] 2024	86 (6wks), 209 (1-year)	Feces	pregnancy through early childhood	x	-	-	x	-	-	-	LR, MICE	-
Shen *et al*. [67] 2024	66 proteins, 71 metabolites, 72 lipids, 34 microsampling, 28 ensure shake study, one 24/7 study	blood, plasma, finger prick microsamples	multiple timepoints, 24/7 study	-	-	x	x	-	-	Lipidomics, Cytokines	LR, WT, ANOVA, NA, SC, CA, JI	x
He *et al*. [68] 2024	three groups (six mice/group)	hippocampal tissues, cecum tissues, serum	cross-sectional study	-	x	-	x	x	-	-	PCA, ANOVA, T-test, SC	-
De *et al*. [69] 2024	six murine models and pediatric patient cohorts	Feces, urine	Months (weekly)	-	-	-	x	x	-	Lipidomics	MOFA, ANOVA, T-tests, CA	x
Brealey *et al*. [70] 2024	140	Gut content, gut tissue and pellets of feed	-	x	x	-	x	x	-	-	PERMANOVA, PCA, LM,WT	-

Abbreviations: AT, Adonis test; BCD, Bray–Curtis dissimilarity; CA, correlation analysis; CHI, chi-squared periodogram analyses; CN, co-occurrence network; Cy, Cytoscape; CV, cross validation; DAMS, Drosophila Activity Monitoring System; DEA, differential expression analysis; DIA-MS, Data-independent acquisition mass spectometry; DIABLO, Data Integration Analysis for Biomarker discovery using Latent cOmponents; DS, descriptive statistics; EN, Elastic-Net; GOE, Gene Ontology enrichment; GR, generalized regression; HC, hierarchical clustering; IPA, ingenuity pathway analysis; KM, K-means clustering; KW, Kruskall–Wallis test; LASSO, Least Absolute Shrinkage and Selection Operator; LC-MS, liquid chromatography-mass spectrometry; LMM, linear mixed models; LR, linear regression; MAASLIN, multivariate correlation analysis based on linear models; MCPT, Monte Carlo Permutation Test; Mh, Megahit; MICE, multiple imputation by chained equations; ML, machine learning; MLR, machine learning regression; MO, Multi-Omics; MOFA, Multi-Omics Factor Analysis; MORE, Multi-Omics Regulation; MT, Mantel Test; MU, Mann–Whitney U Test; NA, NetworkAnalyst; N-PLS, Partial Least Squares regression; NMDS, nonmetric multidimensional scaling; NN, neural network; OGRN, overall gene regulatory network; OPLS-DA, Orthogonal Partial Least Squares Discriminant Analysis; PC, Pearson correlation; PIC, principal interaction contrast; PR, Pearson Regression; RA, Ridge Analysis; RF, Random Forest; ROC, Receiver Operating Characteristic; SC, Spearman’s Correlation; SI, Shannon Indices; T4F, Tax4Fun; TCGSA, time course gene set analyses; t-SNE, t-distributed Stochastic Neighbor Embedding; VCA, Variance Contribution Analysis; VD, Data Visualization; VND, Venn Diagram; WT, Wilcoxon Rank Sum Test.

**Table 2:** Overview of multi-omics “microbiome-free host” studies, including authors, year of study, sample type, temporal sampling frequency, data types (Genomics, Transcriptomics, Proteomics, Meta-bolomics and Others), and applied modeling or ML approaches

Authors (Year)	Number of samples	Sample types	Time-Series (frequency)	G	T	P	MB	Others	Modeling approach	ML
Ansong *et al*. [71] 2013	three	cell cultures	8 h (hourly)	-	x	x	x	Metagenome	microarray analysis, LC-MS, NMR, GC-MS, context likelihood of relatedness, Louvain-community-finding algorithm	x
Kihara *et al*. [72] 2014	15	cell cultures	(hourly)	-	x	-	-	Lipidome	linear kinetics, ODE model	-
Gong *et al*. [73] 2015	four	cell cultures	4 timepoints	-	x	-	-	Epigenome	LR, Bayesian network model	x
Tan *et al*. [74] 2017	eight	cell cultures	16 h (hourly)	-	-	x	-	Phospho-proteome	ANOVA, WCGNA for NA	x
Harvald *et al*. [75] 2017	42	whole organism	16 h (hourly)	-	x	x	-	-	LC-MS, PC, HC, KEGG, GOE	x
Shih *et al*. [76] 2017	1205 AN + 1948 control	blood	-	x	-	x	x	Lipidome	LC-MS, normality tests, ANOVA	-
Ahn *et al*. [77] 2017	27	whole plants	6 h (multiple timepoints)	-	x	-	-	Epigenome	TF networks, Cascade tree, T-test, Fisher’s test	-
Sánchez-Gaya *et al*. [78] 2018	16	cell cultures	-	-	x	-	-	Epigenome	N-PLS, MORE	x
Tasaki *et al*. [79] 2018	hundreds	blood, cell cultures	years (weekly to monthly)	-	x	x	-	-	PLSR	-
Sarigiannis *et al*. [80] 2018	350 children	urine	multiple timepoints	x	x	x	x	Epigenome	Correlation globe plots for assotiations using effect size	-
Abreu *et al*. [81] 2018	171 seedlings, 157 leaf samples	seedlings, leaves	12 h (multiple timepoints)	-	x	-	-	Lipidome	graph-guided fused least absolute shrinkage and selection operator, PCA, PC, NA	x
Sumit *et al*. [82] 2019	four	cell cultures	days (daily)	-	x	-	x	-	PCA, GSEA, TCGSA, maSigPro	x
Pavkovic *et al*. [83] 2019	FA model: 5x2; UUO: 4x4(day 0: 3)	kidney tissue	2 weeks (daily)	-	x	x	-	-	FastQC, STAR/Seqbuster, DESEQ, LDA, PCA	x
Simats *et al*. [84] 2020	37, nine excluded	brain tissue	only once	-	x	x	-	-	PCA, Multiple CIA, regularized Canonical CA	x
Lin *et al*. [85] 2020	six	blood, kidney tissue	days (daily)	-	-	x	-	Phospho-proteome	HC, PCA, T-test, ANOVA, NA	x
Zhao *et al*. [86] 2020	23	urine	18 h (hourly)	-	-	x	x	-	The analysis were performed individually on each ’omics	x
Bernardes *et al*. [87] 2020	14	blood	weeks (daily)	-	x	-	-	Epigenome	UMAP, PCA, HC	x
Wang *et al*. [88] 2020	32	whole heads	2 days (Every 3 h)	-	x	x	-	-	DAMS, two-sided hypergeometric test, CHI	x
Seifert *et al*. [89] 2020	22	tumor tissue	-	x	x	-	-	-	PC, HC, heatmaps, VND, differential expression analysis Fisher’s test	-
Zander *et al*. [90] 2020	tissue samples from more entities	seedlings	hours (minute to hourly)	-	x	x	-	-	GC, RTP-STAr package for gene regulatory network	x
Lam *et al*. [91] 2021	78	blood	-	-	x	x	-	-	Pairwise statistical analysis, ’omics was analyzed individually	x
Tarca *et al*. [92] 2021	133	blood	between 4-7 weeks (2 timepoints)	-	x	x	-	-	LASSO, RF, RR, GR, SVM	x
Suvarna *et al*. [93] 2021	two	blood	weeks (weekly)	-	-	x	x	Lipidome	IPA	x
Yang *et al*. [94] 2021	-	blood	-	-	-	-	-	-	-	x
Brands *et al*. [95] 2021	76 (56 after 1 month), 41 CP	blood	1 month	-	x	-	-	Epigenome	MAASLIN, DIABLO	x
Matsuzak *et al*. [96] 2021	nine per time point	liver, blood	4 h (min)	-	x	x	x	-	DS, PCA, HC, NA	x
Sprenger *et al*. [97] 2021	three per time point	liver tissue, blood	48 h (hourly)	-	-	x	-	Lipidome	ANOVA, PC, c-means clustering, HS, T-test	x
Wu *et al*. [98] 2021	194 (+472 Validation (trauma dataset 2 [TD2]))	blood	days (daily)	-	-	x	x	Lipidome	HC, KM	x
Djeddi *et al*. [99] 2021	36	blood	7 weeks (weekly)	-	x	x	-	-	PCA, T-test	x
Lee *et al*. [100] 2021	three bioreactors	cell cultures	14 days (daily)	-	x	x	x	-	T-test, ANOVA, enrichment analysis, Fisher’s test, HC, PC, heatmap	x
Schwaber *et al*. [101] 2021	one (triplicate samples from one culture)	cell cultures	9 days (daily)	-	x	x	-	-	HC, NA, heatmap	x
Liu *et al*. [102] 2021	60	blood	days (daily)	-	x	x	x	x	HC, N-PLS, UMAP, ANOVA	x
Balzano–Nogueira *et al*. [103] 2021	306	blood	12 months (monthly)	x	x	-	x	Epigenome	NPLS-DA, CV, Partial correlation, GSEA, multi-omics data visualization	x
Sun *et al*. [104] 2021	33	blood	weeks (daily)	-	x	x	x	Lipidome	PCA, functional enrichment analysis, KM, CN, heat map	x
Tang *et al*. [105] 2021	76	blood, urine, fingernails	days (daily)	-	x	-	x	Epigenome, Lipidome	DS, sPLS-DA, RF, Multivariate Analysis, CIT, GLM, ROC	x
Liu *et al*. [102] 2021	14 patients, 12 000 plasma, 57 000 immune cells	blood, cell cultures	2-4 timepoints	x	x	-	-	-	t-SNE, gene expression profiles at different time points	x
Codrich *et al*. [106] 2021	-	cell cultures	(hourly)	x	x	x	-	-	Mutect2, HC	x
Rodrigues *et al*. [107] 2021	-	cell cultures	72h (hourly)	x	x	-	x	-	COSMOS using prior knowledge, PCA, NA, time-dependent gene clustering, flow injection-MS	x
Singhal *et al*. [108] 2021	four	lung endothelial cells, blood	36 days (weekly)	-	x	x	-	-	PCA, heatmap, MU	x
Clark *et al*. [109] 2021	four seedlings per time point	seedlings	8 h (min to hourly)	-	x	x	-	-	DEA, HC, PCA, PoissonSeq, NA, PC, SC, GLMs	x
Camargo *et al*. [110] 2021	eight	leaves, shoot tissue	26 days (daily)	-	x	-	-	-	GSEA, DESEQ, KM, LASSO, discrimination of gene network structures	x
Sacco *et al*. [111] 2022	186	blood	7 days (days)	x	x	x	-	Epigenome	PC, RF	x
Zoran *et al*. [112] 2022	six	blood	2 months (daily to weekly)	-	x	-	x	-	MU, heatmap, GSEA	-
Neogi *et al*. [113] 2022	12	blood	years	-	x	x	x	-	DESEQ, NA, PCA, T-test	x
Song *et al*. [114] 2022	three to eight mouse (total 36 samples) + 36 human heart samples)	heart tissue	days (hourly)	-	x	x	x	-	T-test, ANOVA, MT, WT	-
Li *et al*. [115] 2022	four different hematopoetic cell lines through development	cell cultures	between day 10-14.5 (multiple timepoints)	-	x	-	-	Epigenome	TAD, gene expression and correlation with TF binding motif	x
Unterman *et al*. [116] 2022	18 pbmc samples + 10	cell cultures	pre/post Covid (weekly to monthly)	-	x	x	-	-	Louvain clustering, UMAP, IgPhyML (lineage tree analysis), WT, MU	x
Su *et al*. [117] 2022	209 patients + 457 controls	blood	months (weekly to monthly)	-	x	x	x	-	UMAP, IPA, heatmap, CA, MU, PCA	x
Pekayvaz *et al*. [118] 2022	82	blood, nasal swab	weeks (daily)	x	x	x	-	-	UMAP, Tempora analysis, NA	x
Morilla *et al*. [119] 2022	five	cell cultures	days (daily)	-	x	x	x	-	HC, PC, t-SNE, NN, OGRN, T-test, ANOVA	x
Cui *et al*. [120] 2022	300	whole plants, seedlings	10 days (twice days apart)	-	x	x	-	Epigenome	DIA-MS, GOE, ANOVA, PCA, box plots, heatmaps, violin plots	x
Reimer *et al*. [121] 2022	96	leaves	14 days (weekly)	-	x	-	x	-	Weighted cluster analysis, DIABLO, ANOVA, PCA	x
Zhang *et al*. [122] 2022	tissue samples from more entities	stems, leaves, roots, buds	weeks (days)	x	x	-	x	-	heat maps, gene expressions vs flower stages	x
Allesoe *et al*. [123] 2023	789 (T2 diabetes)	blood	(0, 18 & 36) month	x	x	x	-	-	T-tests, ANOVA, VAE, MOVE	x
Zheng *et al*. [124] 2023	-	Tea leaves inoculated with Pseudopestalotiopsis theae	(0, 1, 3 & 6) day	-	x	-	x	-	Fisher’s test, GEA, multivariate testing	-
Wang *et al*. [125] 2024	Multiple samples	large intestinal tissues from M. fascicularis, cell lines (Caco-2 and HEK293T) and C. elegans were used for cell culture and RNAi	cross-sectional study	-	-	x	x	-	ANOVA, T-tests, KW, SC, PC	-
Ciurli *et al*. [126] 2024	10 Male, 10 Female 18–45 age	Saliva(above Tongue, below Tongue, right cheek)	3x/day	-	-	x	x	-	HC, PCA, SC, WT, sPLS-DA	x

Abbreviations: AT, Adonis test; BCD, Bray–Curtis dissimilarity; CA, correlation analysis; CHI, chi-squared periodogram analyses; CN, co-occurrence metwork; Cy, Cytoscape; CV, cross validation; DAMS, Drosophila Activity Monitoring System; DEA, differential expression analysis; DIA-MS, Data-independent acquisition mass spectometry; DIABLO, Data Integration Analysis for Biomarker discovery using Latent cOmponents; DS, Descriptive Statistics; EN, Elastic-Net; GOE, Gene Ontology enrichment; GR, Generalized Regression; HC, Hierarchical Clustering; IPA, ingenuity pathway analysis; KM, K-means Clustering; KW, Kruskall–Wallis test; LASSO, Least Absolute Shrinkage and Selection Operator; LC-MS, liquid chromatography-mass spectrometry; LMM, linear mixed models; LR, Linear Regression; MAASLIN, multivariate correlation analysis based on linear models; MCPT, Monte Carlo Permutation Test; Mh, Megahit; MICE, multiple imputation by chained equations;MLR, machine learning regression; MO, Multi-Omics; MOFA, Multi-Omics Factor Analysis; MORE, Multi-Omics Regulation; MT, Mantel Test; MU, Mann–Whitney U Test; NA, NetworkAnalyst; N-PLS, Partial Least Squares regression; NMDS, nonmetric multidimensional scaling; NN, Neural Network; OGRN, overall gene regulatory network; OPLS-DA, Orthogonal Partial Least Squares Discriminant Analysis; PC, Pearson correlation; PIC, Principal Interaction Contrast; PR, Pearson Regression; RA, Ridge Analysis; RF, Random Forest; ROC, Receiver Operating Characteristic; SC, Spearman’s Correlation; SI, Shannon Indices; T4F, Tax4Fun; TCGSA, time course gene set analyses; t-SNE, t-distributed Stochastic Neighbor Embedding; VCA, Variance Contribution Analysis; VD, Data Visualization; VND, Venn Diagram; WT, Wilcoxon Rank Sum Test.

**Table 3:** Overview of multi-omics “host-free microbiome” studies, including authors, year of study, sample type, temporal sampling frequency, data types (Genomics, Transcriptomics, Proteomics, Meta-bolomics and Others), and applied modeling or ML approaches

Authors (Year)	Number of samples	Sample type	Time-series (frequency)	G	T	P	MB	MG	MT	Other	Modeling approach	ML
Muller *et al*. [127] 2014	one	wastewater treatment anoxic phase	1 year (monthly)	-	x	x	x	x	-	Meta-proteomics, Meta-transcriptomics	WT	x
Mannan *et al*. [128] 2015	-	bioreactor	-	x	x	x	x	-	-	-	Kinetic modeling	x
Alessi *et al*. [129] 2018	three	compost, wheat straw	8 weeks (weekly)	-	-	x	-	-	x	-	PCA, ANOVA, VND, HC, MDS	x
Han *et al*. [130] 2018	three	bioreactor	12 h (min to hourly)	-	x	x	x	-	-	-	PCA, OPLS-DA	x
Watahiki *et al*. [131] 2019	two	groundwater	3 months (daily to monthly)	x	-	-	-	x	-	-	PCoA, DESEQ, HC	x
Wang *et al*. [132] 2019	one	bioreactor, partial-nitritation anammox reactor	6 months (weekly to monthly)	-	-	-	-	x	x	-	PC, HC	x
Kim *et al*. [133] 2020	one	bioreactor	2 days (minute to daily)	-	x	-	x	-	-	-	HC, PCA, VND, ANOVA, sPLS-DA	x
Delogu *et al*. [134] 2020	pseudo time-series of three flasks per time point	bioreactor	43h (5 h)	-	x	-	x	x	-	Meta-proteomics	Protein expression control analysis, PC, PCA, LMM	x
Breister *et al*. [135] 2020	six	bioreactor	24 weeks (weekly)	x	-	-	-	x	x	-	-	x
Kralj *et al*. [136] 2022	one	bioreactor	-	-	-	x	-	-	-	Lipidomics	T-test volcano plot	x
Kleikamp *et al*. [137] 2023	three wastewater treatment plants	Aerobic granular sludge of 2 mm	-	-	-	x	x	-	-	Lipidomics, Cytokines	KEGG, COG terms, PFAM	-
Dong *et al*. [138] 2024	-	PHE-contaminated soil	28 days	-	-	-	x	x	-	-	Heatmap, NA	-
Delogu *et al*. [139] 2024	51, 21	floating biomass	1.5 years(weekly)	-	x	x	-	x	-	-	LR, CA, Ljung-Box test, Kwiatkowski–Phillips–Schmidt–Shin test	x

Abbreviations: AT, Adonis test; BCD, Bray–Curtis dissimilarity; CA, correlation analysis; CHI, chi-squared periodogram analyses; CN, co-occurrence network; Cy, Cytoscape; CV, cross validation; DAMS, Drosophila Activity Monitoring System; DEA, Differential expression analysis; DIA-MS, Data-independent acquisition mass spectometry; DIABLO, Data Integration Analysis for Biomarker discovery using Latent cOmponents; DS, Descriptive Statistics; EN, Elastic-Net; GOE, Gene Ontology Enrichment; GR, Generalized Regression; HC, Hierarchical Clustering; IPA, Ingenuity Pathway Analysis; KM, K-means Clustering; KW, Kruskall–Wallis test; LASSO, Least Absolute Shrinkage and Selection Operator; LC-MS, liquid chromatography-mass spectrometry; LMM, linear mixed models; LR, Linear Regression; MAASLIN, multivariate correlation analysis based on linear models; MCPT, Monte Carlo Permutation Test; Mh, Megahit; MICE, multiple imputation by chained equations; ML, machine learning; MLR, machine learning regression; MO, Multi-Omics; MOFA, Multi-Omics Factor Analysis; MORE, Multi-Omics Regulation; MT, Mantel Test; MU, Mann–Whitney U Test; NA, NetworkAnalyst; N-PLS, Partial Least Squares regression; NMDS, nonmetric multidimensional scaling; NN, neural network; OGRN, overall gene regulatory network; OPLS-DA, Orthogonal Partial Least Squares Discriminant Analysis; PC, Pearson correlation; PIC, principal interaction contrast; PR, Pearson Regression; RA, Ridge Analysis; RF, Random Forest; ROC, Receiver Operating Characteristic; SC, Spearman’s Correlation; SI, Shannon Indices; T4F, Tax4Fun; TCGSA, time course gene set analyses; t-SNE, t-distributed Stochastic Neighbor Embedding; VCA, Variance Contribution Analysis; VD, Data Visualization; VND, Venn Diagram; WT, Wilcoxon Rank Sum Test.

**Table 4:** Overview of multi-omics method studies, detailing statistical and ML approaches (univariate/multivariate analysis, network analysis, DL, supervised and unsupervised ml, mechanistic models), along with predictive performance, interpretability, ease of use, and activity status of development or maintenance

Authors(Year)	Uni/multivariate	Network analysis	Deep learning	Other supervised ML	Ordination or unsupervised ML	Mechanistic model	Predictive performance	Interpret-ability	Ease of use	Activity of development/Maintenance
Gibbs *et al*. [140] 2014	-	x	-	-	-	-	2	2	2	deprecated
Bodein *et al*. [141] 2019	-	-	-	x	x	-	3	2	2	active
Chong *et al*. [142] 2019	x	x	-	x	x	-	3	3	3	active
Chung *et al*. [143] 2019	x	-	x	-	x	-	3	2	2	inactive (3 years)
Williams *et al*. [144] 2019	x	-	-	-	-	-	3	3	3	active
Oh *et al*. [145] 2020	-	x	x	-	-	-	2	3	2	unknown
Conard *et al*. [146] 2021	x	x	-	-	x	x	3	3	3	sporadic activity
Liu *et al*. [102] 2021	-	-	x	-	-	-	3	1	1	inactive(1year)
Mallick *et al*. [147] 2021	x	-	-	x	x	-	2	3	3	active
Ruiz-Perez *et al*. [148] 2021	-	x	-	-	-	-	3	2	1	inactive (1 year)
vanRiel *et al*. [149] 2021	-	-	-	x	-	x	2	2	2	inactive (8 years)
Anžel *et al*. [150] 2022	x	-	x	-	x	-	1	3	3	active
Bodein *et al*. [151] 2022a	-	-	-	x	x	x	2	3	3	active
Bodein *et al*. [152] 2022b	x	x	-	x	x	-	2	3	3	inactive (1 year)
Hamzeiy *et al*. [153] 2022	-	x	-	-	-	-	3	3	3	active
Abe *et al*. [154] 2023	x	-	-	-	x	-	2	3	3	active
Allesoe *et al*. [123] 2023	x	-	x	-	x	-	2	3	3	active
Mallick *et al*. [155] 2024	x	-	-	x	-	x	2	2	3	active

## Results

**Figure 3:**
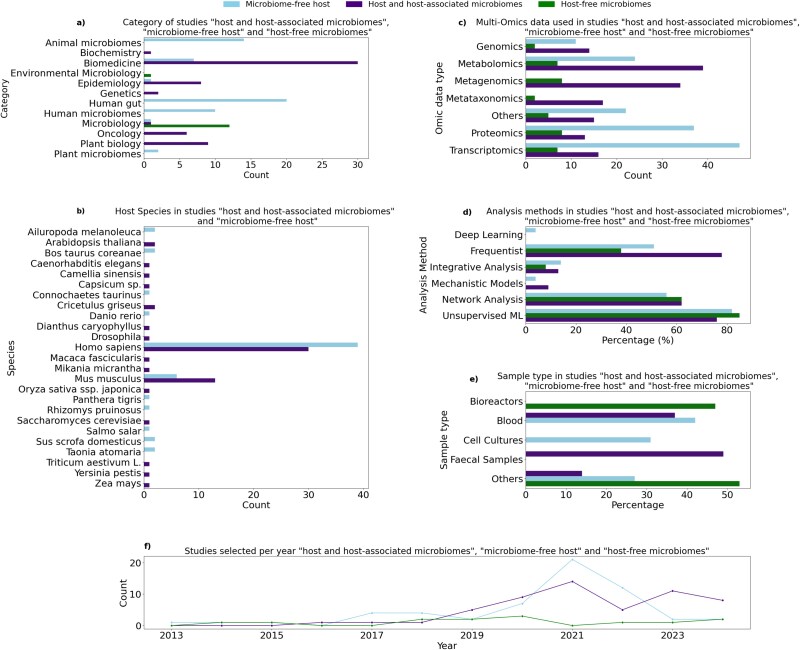
Comparative breakdown of longitudinal studies from 2013 to 2024. The analysis captures (a) distribution of study types including “host and host-associated microbiome,” “microbiome-free host,” and host-free microbiome” studies, (b) diversity of host species studied across the dataset in “host and host-associated microbiome,” and “microbiome-free host” studies, (c) omics data types most frequently used (e.g. transcriptomics, metagenomics, metabolomics) in “host and host-associated microbiome,” “microbiome-free host,” and host-free microbiome” studies, (d) analytical methods applied, showing prevalence of classical versus DL models in “host and host-associated microbiome,” “microbiome-free host,” and host-free microbiome” studies, (e) sample types collected (e.g. blood, fecal, tissue) in “host and host-associated microbiome,” “microbiome-free host,” and host-free microbiome” studies, and (f) publication trends over the past decade in “host and host-associated microbiome,” “microbiome-free host,” and host-free microbiome” studies. Notably, DL remains underutilized in this domain despite increasing data availability, highlighting a potential area for future methodological advancement.

**Figure 4:**
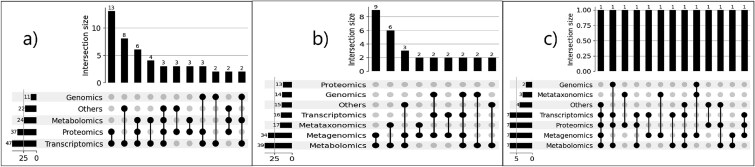
Upset plot visualizing the overlap of multi-omics data type across the reviewed studies: (a) “microbiome-free host” with intersection size = 2, (b) “host and host-associated microbiome” with intersection size = 2, and (c) “host-free microbiome” with intersection size = 1.

Our systematic review highlights the diversity of multi-omics literature in terms of research focus and methodology. We categorized the reviewed studies into “host and host-associated microbiome,” “microbiome-free host,” and “host-free microbiome” data (Fig. 3; Table 1, Table 2, Table 3). These categories summarize the distribution of categories, sample types, data types, host species, and analysis methods. Figure 4 represents the overlap of multi-omics data type across the reviewed studies.

Overall, the types of multi-omics data and associated computational methods in these studies ranged from general exploratory techniques to more advanced time-series-specific methods designed for longitudinal datasets. Common study designs for longitudinal studies included monitoring studies, cohort studies, and intervention studies, each suited to different question:

- Cohort studies track a sample or cohort of randomly selected individuals from a homogeneous group over time, frequently documenting the progression of the disease or natural variability. These studies are useful for finding patterns or biomarkers linked to certain outcomes, including the start or recovery from illness [27, 50, 98].
- Monitoring studies involve the ongoing or sporadic monitoring of participants for an extended period of time, frequently in uncontrolled or natural settings. Understanding the impact of changes in the environment or lifestyle variables is made easier by such studies [79].
- Intervention studies compare the time-series data across two or more groups undergoing different treatments, such as clinical trials or dietary interventions. These studies are especially helpful for determining how certain therapies affect multi-omics profiles over time [48, 50, 76, 95, 117, 139].

### Methodological frameworks

We identified in total 18 studies that described modeling frameworks for multi-omics time-series analysis (Table 4). The most common analysis method categories included in the frameworks are ordination or unsupervised machine learning (ML) (10 studies), frequentist uni/multivariate methods (10 studies), supervised and network analysis (seven studies), each. We evaluated the methods based on three key aspects: performance, interpretability, and ease of use. These aspects were evaluated with scores using a qualitative score (1 = worst to 3 = best):

- Performance: 3 = strong benchmarking and generalizability; 2 = moderate validation; 1 = minimal evidence.
- Interpretability: 3 = highly transparent outputs; 2 = partially interpretable; 1 = black-box approach.
- Ease of use: 3 = well-documented and maintained code; 2 = limited documentation; 1 = obsolete or unsupported implementation.

Based on this qualitative assessment, we noticed shortcomings in one or more of these criteria in two studies, whose repositories have been deprecated or did not received updates since 2014. The methods developed by Chong *et al*. [142], Conard *et al*. [146], and Hamzeiy *et al*. [153] achieved maximum scores; all of these methods are still actively maintained. The methodology developed by Chong *et al*. [142] showed the broadest application in multi-omics time-series analysis [156–160]. Other methods were frequently cited, but their usage and applications were not clearly described. This indicates a potential gap in reporting or less-defined roles in practical analyses. Interestingly, some of the methods appeared to have been available for a long time before the study itself was published. For example, the code repository of ADAPT (Analysis of Dynamic Adaptations in Parameter Trajectories; van Riel *et al*. [149]) was last updated in 2014, while the study was published in 2021.

### Multi-omics data integration

Although including multiple ’omics layers offers advantages in microbiome research, the consensus on the optimal approach to achieve the integration of different ’omics layers in the inference framework is yet to be achieved. Some studies use many different aspects of microbiome observations, such as *meta-genomics*, *meta-transcriptomics*, and *meta-proteomics* data, capturing different aspects of the biological processes (e.g. taxonomic, potential, and realized function). Integration approaches include

- using different ’omics layers to capture complementary information about biological processes;
- performing batch correction or normalization across multiple ’omics before integration;
- concatenating data from different ’omics into a single matrix for downstream analysis; and
- separate analysis: rraditionally, each ’omics layer has been analyzed independently, followed by qualitative comparison of parallel changes.

### Dimensionality reduction

Dimensionality reduction is commonly used as the initial stage of most of the multi-omics studies, because it enables exploratory analysis and visualization of the dataset. Common methods include the following: **Principal component analysis (PCA):** a widely used method for dimensionality reduction, which is frequently applied to individual ’omics datasets to extract features or reduce noise prior to integration [18, 20, 22, 23, 25, 27, 29, 30, 35, 37–40, 42, 45, 46, 48, 51, 63, 68, 70, 81–85, 87, 95, 96, 99, 104, 107–109, 113, 117, 120, 121, 126, 129, 130, 134, 161]. For basic integration, some methods employ PCA immediately after concatenating abundance tables from many ’omics (such as *transcriptomics* and *proteomics*) into a single matrix. Pipelines have been shown to use PCA to find latent features for downstream classification tasks using combined *meta-genomics* and *meta-bolomics* data [162]. **Principal coordinate analysis (PCoA):** it is essentially a form of classical multidimensional scaling (MDS) that extends PCA to non-Euclidean dissimilarity measures and a common choice in microbiome research. This was the second most used method in the studies included in this review [28, 29, 31, 34, 37, 45, 47, 51, 62, 64, 131]. In time-series multi-omics, these methodologies enable temporal trajectory studies by condensing data variance between time points into a reduced number of interpretable dimensions. MDS has also been employed in multi-omics integration by computing joint dissimilarity metrics across ’omics layers, though this may require careful normalization to balance feature scales. **Other nonlinear methods:** they have also been used, including Isomap, t-SNE [35, 58, 102, 119] and UMAP [57, 87, 102, 116–118]. While these are often applied to single-omics data, recent workflows integrate multi-omics by first reducing each layer separately using PCA and then aligning embeddings. For example, Compound-SNE aligns t-SNE projections from multiple single-cell ’omics datasets while preserving sample-specific structures [163]. Such methods address the limitations of naive concatenation by leveraging shared variance or feature-grouping strategies. In summary, while PCA, MDS, or t-SNE are frequently applied to individual ’omics layers, we also identified their use in multi-omics integration, either through concatenation or coordinated embeddings.

### Correlation analyses

The choice of the methods is often based on specific data characteristics, including the type of data (e.g. continuous or categorical), data distribution, and measurement scale. For example, pairwise correlation coefficients have been calculated between relative abundances of microorganisms and the expression levels of several genes. Popular approaches include the following: **Pearson’s correlation:** in longitudinal multi-omics, such correlations can be used to monitor the evolving associations between molecular variables across time, aiding in the identification of consistent or temporary interactions across temporal points. Pearson’s coefficient has been applied in several studies to monitor evolving associations across time points. [38, 44, 75, 81, 89, 97, 100, 109, 111, 119, 132, 139]. **Spearman correlation:** a rank-based measure that captures monotonic relationships, often used when data may not be linear. Spearman’s correlation has been used in many longitudinal multi-omics studies to detect nonlinear trends. [40, 43, 45, 49, 59, 61, 64, 67, 109, 125, 126] **Compositional data considerations:** the possibility of compositionality bias must be taken into account when determining correlations for compositional data, such as relative abundances of microbiomes. By definition, compositional data add up to a constant (100% relative abundance), therefore modifications to one component always impact the others. Pearson and Spearman correlations are subject to this bias, unless specifically corrected for it [164]. The centered log ratio (CLR) transformation is often used to mitigate compositional effects by converting compositional data into a log ratio space. The CLR transformation calculates the logarithm of the abundance of each trait relative to the geometric mean of all traits in the sample in order to mitigate dependencies between features [165]. Whereas the use of CLR or comparable transformations is increasingly recognized as standard practice in microbiome research to ensure reliable correlation analyses, this issue was not always addressed in the reviewed studies. **Canonical correlation:** it is an extension of the PCA to multiple datasets [166, 167], which can quantify multivariate correlations between datasets. It can identify correlated feature sets in paired datasets, instead of individual correlated pairs of individual features detected by the standard Pearson and Spearman cross-correlation. This approach has been recently used, e.g. by Revilla *et al*. [41] and Simats *et al*. [84].

### Clustering and similarity network methods

Clustering methods are used to discern general patterns in a dataset. Clustering can be performed on both samples and features. Key approaches include the following: **Clustering with dissimilarity measures:** traditional clustering using Euclidean distance, Manhattan distance, or Bray–Curtis dissimilarity has been applied in some studies to perform clustering across samples [22, 23, 47, 50, 62]. Clustering techniques can be used for basic integration by defining distance metrics across several multi-omics layers. Integrative techniques that are relevant to multi-omics time-series analysis include iCluster [168] and Similarity Network Fusion (SNF) [169]. These methods are designed to handle the complexity of multi-omics data by capturing both shared and layer-specific patterns across time. **SNF:** it is a powerful integrative method that constructs and fuses sample similarity networks across multiple ’omics layers. SNF functions in the sample space, as opposed to feature-based networks, where nodes indicate samples (such as patients or time points) and edges indicate pairwise similarities between samples according to their ’omics profiles. To create a unified representation that captures both shared temporal patterns (common across ’omics layers) and layer-specific temporal patterns (unique to a particular ’omics layer), SNF builds distinct similarity networks for each ’omics layer (e.g. *transcriptomics*, *proteomics*) at each time point. This fusion approach is especially useful for detecting dynamic biological changes that are consistent across various data types because it makes use of local commonalities and complementary information across ’omics layers. In cancer research, Wang *et al*. [170] used SNF to integrate data on mRNA expression, DNA methylation, and miRNA expression. This has revealed temporal trajectories and clinically significant subgroups that were not visible in individual ’omics layers. **iCluster:** it concentrates on separating the data into shared and distinct patterns that show temporal dynamics, such as metabolites, which only show up at particular times, or gene expression levels, which fluctuate over time. iCluster thus finds sample clusters that change over time in response to biological disturbances, such as the course of a disease or the results of therapy, by modeling these time-specific properties. Shen *et al*. [171] utilized iCluster as a joint latent variable model that combines transcriptomic, proteomic, epigenomic, and genomic data to categorize tumor subtypes. iCluster outperformed conventional separate clustering techniques.

### Regression and classification

Regression and classification techniques can be used for asymmetric quantification of associations between two ’omics, for instance to predict values of one set based on the other. **Linear mixed models and regression:** many studies have focused on the case of a single ’omics regression (i.e. predicting one ’omics layer as the response from another as the predictor, $y \sim x$). However, an equally important and emerging direction involves integrating two or more ’omics layers to predict external covariates such as age, BMI, or overall health. In the simplest approach, datasets from different ’omics layers can be concatenated. Several studies used linear mixed models [19, 20, 22, 28, 50, 51, 56, 58–60, 134] to establish associations between ’omics layers. Several studies have explicitly applied multi-omics regression and classification approaches in time-series settings. For instance, studies integrating *meta-genomics* and *meta-bolomics* datasets have concatenated data to predict host phenotypes over time, thereby revealing dynamic associations that evolve with aging or health status [37, 44]. **Classification (LDA and sPLS-DA):** for classification, several studies used linear discriminant analysis (LDA) [30, 34, 43, 56, 83]. This method identifies the most optimal hyperplane to separate labeled samples. Also, extended discriminant analysis method called sparse variant (sPLS-DA) was used in several studies [23, 39, 40, 46, 47, 49, 105, 126, 133]. sPLS-DA performs variable selection and classification in a one-step procedure and enables the selection of the most predictive or discriminative features in the data to classify the samples. Several studies utilized individual ’omics even within multi-omics studies. However, recent studies have extended these classification approaches to directly integrate multiple ’omics layers. In such frameworks, sPLS-DA has been successfully used to track time-evolving discriminative features across *transcriptomics*, *proteomics*, and *meta-bolomics* data, thus enhancing the predictive and interpretative power in longitudinal studies [47, 126]. Linear mixed models efficiently incorporate random effects related to temporal variability, thereby enabling researchers to rigorously evaluate longitudinal trends in multi-omics associations.

### Temporal modeling and longitudinal data analysis

Various methods have been specifically devised for longitudinal multi-omics. One of the main challenges in multi-omics integration is handling asynchronous sample intervals and disparate progression rates across various ’omics layers. **Dynamic Bayesian Networks (DBNs):** it is especially useful in this context, as they determine directed connections among biological entities—such as host genes, metabolites, and microbial taxa—while capturing the nonlinear and conditional dependencies present in biological systems. **Vector autoregressive models:** state-space models and VAR models assume linear relationships over time and have been used in multi-omics. These methods capture temporal dependencies but may be limited if underlying dynamics are nonlinear. **Recurrent neural networks (RNNs):** they can achieve high prediction accuracy; they frequently operate as “black boxes” that lack interpretability. Ruiz *et al*. [148] addressed these challenges by proposing the PALM pipeline. This approach first aligns longitudinal data from host *transcriptomics*, *meta-bolomics*, and *meta-genomics* and then uses DBNs to reconstruct a unified interaction network. The PALM pipeline has effectively identified both known and novel metabolite–taxon interactions in patients with inflammatory bowel disease, with experimental validation further supporting these findings. Other approaches to modeling temporal dependencies across ’omics layers include state-space models [172] and vector autoregressive models [173]. Certain variants of RNNs, such as long short-term memory (LSTM) networks, are capable of capturing temporal patterns in multi-omics data [174]. Additionally, network-based methods—including temporal correlation networks and multilayer networks—can combine and examine patterns in multi-omics time-series, emphasizing the dynamical transitions and trends [152].

### Neural networks and deep learning

The integration of heterogeneous multi-omics data collected over time has been enabled by recent advances in deep learning (DL), providing unprecedented insights into biological systems and disease processes [14, 175, 176]. **Recurrent architectures (gated recurrent units):** Jain and Safo [177] developed a DL pipeline that uses gated recurrent units to extract time-dependent features for disease classification, thereby integrating cross-sectional and longitudinal multi-omics data, including *transcriptomics*, *meta-bolomics*, and *meta-genomics*. It stands out for its ability to handle nonoverlapping samples and variable-length time-series data, maximizing the use of available heterogeneous datasets. **Graph neural networks (ConvGNN):** ConvGNN framework for multi-omics categorization of chronic obstructive pulmonary disease (COPD) was established by Zhuang *et al*. [178] as a complementary method. Unlike traditional classifiers, this study improves prediction accuracy by combining protein–protein interaction networks from known databases with longitudinal proteomic and transcriptomic data. The ConvGNN technique improves the interpretability and efficiency of COPD classification models by integrating biological network information into the learning process. **Disease-Atlas:** Lim and van der Schaar [179] introduced Disease-Atlas, a DL technique that simultaneously models time-to-event outcomes and longitudinal data. This method enables more accurate predictions of disease progression by using adaptive neural network architectures to capture the dynamic evolution of disease states from multi-omics inputs.

### Multi-omics (latent) factor analysis

Multi-omics factor analysis (MOFA) is a powerful framework designed to separate variation in complex multi-omics datasets by providing a shared low-dimensional representation that captures common and modality-specific signals. Argelaguet *et al*. [180] used cross-sectional cohort of chronic lymphocytic leukemia patient samples, where MOFA integrated somatic mutations, RNA expression, DNA methylation, and *ex vivo* drug responses to uncover major dimensions of disease heterogeneity (such as immunoglobulin heavy-chain variable region status and trisomy of chromosome). MOFA has proven invaluable for revealing underlying biological processes in complex multi-omics datasets. Several studies have extended MOFA to address the difficulties presented by longitudinal data based on this foundation. Zimmer *et al*. [35] analyzed longitudinal multi-omics data including *proteomics*, *meta-bolomics*, microbiomes, and clinical laboratory values, using the Pareto Task Inference (ParTI) approach. This method showed that three wellness stages and one aberrant health condition were defined by the mapping of clinical lab data onto a tetrahedral structure. Similarly, MOFA was used by De *et al*. [69] on a longitudinal murine model. Their analysis revealed that gut microbial and metabolic alterations, particularly in bile acid, energy, and tryptophan metabolism, preceded allergic inflammation following $\beta $-lactoglobulin sensitization. These findings were validated in children with IgE-mediated cow’s milk allergy (IgE-CMA), linking gut dysbiosis to early immune responses. This highlights microbial and metabolic markers as potential early predictors of IgE-CMA. **MEFISTO:** Gaussian process regression is integrated into MEFISTO to model spatio-temporal dependencies in longitudinal multi-omics data, extending traditional factor analysis frameworks. In their foundational work, Velten *et al*. [181] applied MEFISTO to evolutionary developmental atlases (gene expression data from five species across organ development), longitudinal microbiome studies (43 children over two years), and single-cell multi-omics datasets (mouse gastrulation with RNA, methylation, and chromatin accessibility). These applications revealed conserved developmental trajectories, species-specific variation, and dynamic gene regulation, outperforming conventional methods in imputing missing data and aligning temporal patterns across misaligned groups. **MOFA+:** The framework underpinning MEFISTO extends these capabilities to integrate multimodal single-cell data across diverse sample groups. MOFA+ has been used to model heterogeneity in immune-mediated diseases by jointly analyzing DNA methylation, chromatin accessibility, and transcriptomic profiles, identifying latent factors linked to dynamic T cell activation states [182]. This approach leverages computationally efficient variational inference to unify large-scale and single-cell datasets, enhancing patient stratification through temporal or disease progression-associated features.

## Discussion

### Time-series data collection

Microbiomes are inherently dynamic; therefore, gathering and analyzing longitudinal data is necessary to better understand the interactions within host-microbiome communities. Such studies can help us to better understand complex mechanisms between the multi-omics profile of an organism and its phenotype, as well as how biological systems respond to variations in their genetic makeup or external environments. Including multiple samples in the analysis allows us to identify the essential core interactions between a host and its microbiome. This approach also provides a unique opportunity to quantify the correlation or divergence between time points and compare these metrics across the different layers of ’omics. Collecting time-series data in the context of multi-omics poses distinct and considerable challenges. Obtaining consistent sampling across these domains at regular intervals is especially challenging when investigations span extended periods. Moreover, regulating environmental and experimental variability is difficult due to the dynamic nature of living systems. Gene expression, protein synthesis, and metabolic activity can change unpredictably, even under steady conditions, causing variability that can hinder data interpretation. Researchers are thus increasingly implementing stringent criteria for sample handling, storage, and archiving to ensure uniformity over time and between study sites [183]. Ethical and logistical constraints introduce additional complications, particularly in studies involving humans or animals. Repeated sampling may be impractical due to ethical considerations or the intrusive nature of the methods. To overcome these obstacles, researchers frequently employ “pseudo time-series” approaches by sampling distinct individuals (yet if possible similar in the major characteristics) at various time intervals and merging the data to deduce temporal trends [184, 185]. While this can provide useful insights, it cannot match the depth of information obtained by monitoring changes within the same individual over time. Consequently, it may overlook nuanced biological rhythms or fail to adequately document the comprehensive development of diseases. Designing an efficient time-series study requires achieving a careful balance among sampling frequency, temporal resolution, and the ethical constraints associated with the study’s subjects and aims. By integrating host and microbiome data across multiple time points into a unified framework, we can maximize its potential. This approach enables our understanding and accurate prediction of dynamic phenotypic traits, including growth dynamics, health, drug response, disease susceptibility, and pathogenesis [173].

### Data types and data structures

Multi-omics data are often sparse due to many practical and ethical challenges related to experimental design and sample collection. A significant issue is the lack of one-to-one matching across different ’omics layers, meaning that not all samples are measured across all modalities (e.g. *genomics*, *transcriptomics*, *proteomics*). This results in unevenly distributed and missing data, which can limit the robustness of conclusions drawn from such datasets. A typical multi-omics study might examine six major technique categories: *genomics*, *transcriptomics*, *proteomics*, *meta-bolomics*, *epigenomics*, and single-cell ’omics. However, due to technical limitations, cost, or sample availability, only a subset of these techniques is often applied, leading to incomplete data integration and potential biases in analysis. Hence, it weakens the opportunity of comparing different studies since they collect different type of data. The arrangement of data in appropriate containers and formats plays a significant role in managing multi-omics time-series datasets. Efficient data storage and retrieval technologies provide tools to readily access, process, and analyze data across different ’omics layers. Contemporary data storage formats, such as HDF5, OME-Zarr [186], OME-NGFF [187], have emerged as favored choices due to their capacity to manage extensive, multidimensional datasets effectively [188]. The R/Bioconductor community has advanced statistical data analysis methods based on specific multi-assay data structures [189, 190]. These and other formats support multisource data integration and can facilitate hierarchical data organization, permitting researchers to consolidate many types of ’omics data within a singular container while preserving their unique structures and formats. Moreover, multi-omics data retrieval tools (e.g. HoloFoodR [191]) and interactive applications (e.g. iSEEtree [192]) support the exploration and analysis of longitudinal and other multi-omics datasets based on such data structures. Interoperability across diverse data formats and platforms is especially crucial in multi-omics research, as it enables for smooth integration of datasets from different sources or studies. Standardized formats like JSON and XML for metadata annotation assist in maintaining compatibility, enabling researchers to correlate data on gene expression, protein levels, metabolite concentrations, and other factors across time points. This interoperability is crucial in collaborative studies with multisite or multidisciplinary teams that contribute data to a common repository. The utilization of modular and adaptive data containers facilitates data accessibility, retention, and reproducibility, thus facilitating deeper insights into host-microbiome interactions over time.

### Underutilized analysis techniques

Mechanistic modeling of multi-omics measurements holds the promise of providing a more comprehensive and nuanced representation of biological systems, when compared with data-driven inference and DL methods. Mechanistic models, such as dynamic models employing differential equations, or agent-based models could encapsulate key aspects of a system’s behavior [193]. Such approaches have been previously used to elucidate molecular interactions, gene regulatory networks, and causal linkages [194]. Moreover, they have demonstrated utility in uncovering regulatory mechanisms in both healthy and pathological conditions, as well as in examining recovery processes from disrupted states [146]. Thus, mechanistic models can help establish a solid basis for refining interactions, assessing and validating ranges of kinetic parameters, identifying most important model components, and to better understand the underlying mechanisms and drivers of microbiome dynamics. The computational strategies for integrating longitudinal multi-omics data are only starting to emerge. As previously highlighted, separate analysis and post hoc comparisons of multi-omics data are often inadequate for gaining deeper insights into the interactions between the different ’omics layers. Integrative techniques are essential for understanding interactions across diverse biological processes and ’omics data. The intrinsic variability and irregular data availability multi-omics time-series underscore the necessity for adaptability in analytical frameworks. Whereas traditional methods often presume comprehensive and uniformly distributed data, biological data often display deficiencies or uneven temporal intervals due to logistic limitations or sample attrition [195]. Adaptive techniques that can tackle these issues are crucial for producing significant discoveries. Methods like imputation of absent values, interpolation models, Bayesian techniques, and ML algorithms have arisen as essential instruments in this domain. ML techniques, including RNNs [196, 197], LSTM models [143], and transformers [198, 199], show great potential for modeling temporal correlations in multi-omics data, even when faced with missing or irregularly spaced observations. While transformers have not yet been extensively applied to time-series data (to the best of our knowledge), their inherent memory mechanisms and ability to capture long-range dependencies suggest they could be highly effective for modeling such data in the future. Nonetheless, these models frequently need substantial computational resources and specialized knowledge, which may restrict their wider utilization. Furthermore, the amalgamation of diverse data types in time-series analysis continues to provide a significant difficulty. Each ’omics data type displays unique properties to consider. Thus, enhancing the adaptability of analytical tools will be essential for realizing the complete potential of longitudinal multi-omics. As these databases grow in complexity and scale, adaptive methods will be essential for enabling comprehensive analyses.

### Real-world applications of multi-omics time-series analysis

Recent findings underscore the significant uses of multi-omics time-series analysis in microbiome research. Lloyd-Price *et al*. [22] utilized *metagenomics*, *meta-transcriptomics*, *proteomics*, and *metabolomics* time-series data in Inflamatory Bowel Disease patients, revealing microbial and metabolic alterations that precede disease intensification. In other study, Hagan and Cortese [200] integrated longitudinal microbiome, *transcriptomic*, and *metabolomic* data in vaccination research, demonstrating that gut dysbiosis impairs antibody responses to influenza vaccines. In obesity therapies, Mohr *et al*. [201] characterized gut microbiota and plasma metabolites longitudinally, correlating microbial and metabolic characteristics with weight-loss results across various diets. These empirical instances illustrate how longitudinal multi-omics might uncover dynamic, predictive biomarkers across many health problems.

### Adoption gap

Despite the availability of the proposed methods, their integration into widely utilized computational frameworks remains limited. In many cases, either the implementations are unavailable, or they are restricted to specific software environments that may not be accessible to all researchers. Furthermore, many methods are tailored to particular use-cases, making them challenging to adapt for other types or collections of data. This has resulted in an “adoption gap” of the new methods. Cross-disciplinary training programs could support the broader computational application and development of skills among applied researchers. The creation of intuitive graphical user interfaces and streamlined workflows in widely used platforms and cloud-based technologies might further enhance the adoption of these methods.

**Figure 5:**
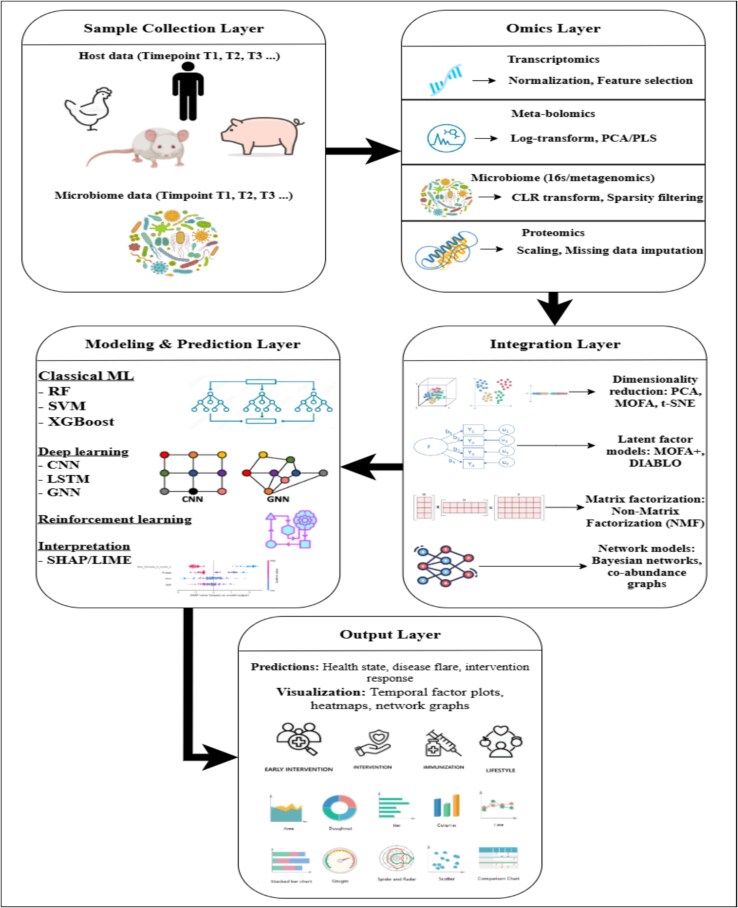
Overview of the end-to-end multi-omics data science pipeline for predictive modeling in longitudinal host–microbiome studies. The pipeline is composed of five core layers: (1) **Sample Collection Layer** gathers temporal host and microbiome data across multiple species (e.g. humans, mice, pigs), (2) **Omics Layer** processes various data modalities including *transcriptomics, proteomics, meta-bolomics, and microbiome* data through transformation and normalization steps, (3) **Integration Layer** combines heterogeneous omics datasets using techniques such as dimensionality reduction (PCA, MOFA), latent factor models (DIABLO), and network-based methods (Bayesian networks), (4) **Modeling and Prediction Layer** applies classical ML (e.g. Random Forest, XGBoost), DL (CNN, LSTM, GNN), and interpretation methods (SHAP/LIME) for robust modeling, and (5) **Output Layer** generates predictions (e.g. health state, disease flare) and data visualizations including temporal factor plots, heatmaps, and network graphs.

### Challenges and limitations in multi-omics data integration

The interactions between the biological processes of the host and their microbiome are still only superficially understood. Integrative analysis of the (meta) genomes, (meta) transcriptomes, and (meta) metabolomes of the host and its microbiomes is a more extensive approach than analyzing each of these ’omics data separately [202]. Creating a comprehensive framework that combines data from many ’omics layers and time intervals enables more effective discovery of biological pathways that link, e.g. genomic variation to phenotypic variance. By cross-comparing different ’omics layers, we can examine direct interactions within these layers (e.g. host genome to metabolome) and between the host and its microbiomes. This allows us to comprehensively and systematically understand the intricate biology that underlies the connections between the host genome and health, as well as the composition or diversity of the microbiome [203]. Several limitations and biases in the reviewed studies and their methodologies remain despite the potential of multi-omics integration. Some of the main challenges include the relatively small number of time points, which may be further unevenly spaced or unmatched between different data types, high individual variability, and subject drop-outs [141]. Sometimes it is not possible to collect longitudinal data, e.g. because the sample is drawn from tissue or an organ that is surgically removed. Moreover, invasive sample collection at more than one time point might not be ethically justified and there are high costs involved with sampling at multiple time points. In the case of laboratory animals, it is possible to collect samples that require euthanizing; however, this design does not allow samples from multiple time points to be collected. In these cases, a so-called “pseudo time-series” can be assembled from multiple cross-sectional datasets so that, e.g. disease progression is preserved (see, e.g. [16]). This means that at each time point, the disease state is carefully identified and the full dataset consists of ordered time points that simulate disease progression. Pseudo time-series can thus approximate the collection of true time-series data in these cases. However, intra-individual differences might disguise patterns related to disease progression. A large number of studies include only a few entities that were tracked over time, especially in the context of “host and host-associated microbiomes.” For example, the two studies on swine (*Sus scrofa domesticus*) microbiomes sampled only three [24], or six [204] animals over time. Furthermore, the main problem is generally not limitations in sample size per se but the level of heterogeneity. High data sparsity in multi-omics studies, especially in longitudinal microbiome datasets, poses risks for reproducibility. Sparse measurements may mask temporal associations, inflate variance, and lead to unstable feature selection. For instance, a study applying time-aware PCA to infant gut microbiome data showed divergent patterns when re-evaluated using complete-case analysis, demonstrating sensitivity to missing values. Standardizing imputation and reporting sample coverage will be essential to improving reproducibility.

### Toward a standardized framework for longitudinal multi-omic integration

To conduct longitudinal multi-omic study, we outline a modular workflow presented in Fig. 5 that integrates key steps from sample selection to biological interpretation. This step-by-step strategy takes into consideration the time-dependent complexity of biological systems and the technological differences across omics data. We provide a set of rules to help researchers with every step of the process, from planning the study to getting useful results. First, it is important to get samples from both the host and the microbiome at the same biological time points, making sure that the time resolution matches the biological events being studied (such the course of a disease or the start of a treatment). Second, each omic layer should have its own data preprocessing. For example, *transcriptomic* data usually need to be normalized and have features selected; *meta-bolomics* data need to be log-transformed and have their dimensions reduced; microbiome profiles often need to have their composition changed (e.g. with CLR transformation) and have their sparsity filtered; and *proteomics* data may need to be scaled and have missing values filled in. Third, integrated analysis needs strong computational methods that can deal with noise that is distinct to each modality and has a lot of dimensions. This includes dimensionality reduction (like PCA and MOFA), latent factor modeling (like DIABLO), and network-based methods to find hidden biological signals and connections between different types of data. Fourth, the choice of predictive modeling should depend on the research issue and the data that are available. Classical ML approaches (such random forests and SVMs) or DL models (like CNNs and GNNs) should be used when they are applicable. We need to use methods like SHAP or LIME to turn prediction signals into biological understanding and we need to make sure that model interpretability is a top priority. Finally, specialized visualizations such as heatmaps, network graphs, and temporal factor plots should be used to put the results in context and help people understand and talk about them. This systematic, modular methodology gives longitudinal multi-omics research a solid base that can be used and expanded. It also shows a clear way to improve our understanding of how biological processes change over time at the systems level.

### Implications for future research

In summary, an interdisciplinary data integration strategy should be used to support a better understanding of hierarchically structured complex biological systems. This would enable predicting trajectories of change, optimizing the predictive power of theoretical models and developing successful practices for agriculture, aquaculture, veterinary science, and human health. A better understanding of genotype–phenotype associations, as well as the biological pathways between them, will allow us to identify better interventional targets in biological systems, such as better probiotics in food production systems within agriculture and aquaculture or gene targets for drugs. It will also allow us to develop precision medicine and predict future changes in the microbiome in response to such treatments [205, 206]. Emerging ML paradigms such as reinforcement learning (RL) and explainable AI (XAI) have not yet been widely applied in microbiome multi-omics. RL offers potential for adaptive time-point modeling in response to feedback (e.g. treatment response modeling), while XAI techniques (e.g. SHAP, LIME) can be used to interpret predictions from complex models like neural networks. Incorporating these tools could address long-standing concerns around interpretability in DL workflows.

**Key Points**

- Time-series multi-omics studies are becoming the standard for studying temporal and functional aspects of host-microbiome systems.
- Most studies use only exploratory analyses for summarizing time-series multi-omics data.
- Only a few integrative frameworks exist for analyzing time-series multi-omics data.
- This study presents an overview of the current methods and techniques, thus providing a pipeline for time-series studies starting from data collection to integrative inferences.

## Data Availability

All the code and data tables used for the figures in the manuscript are available on GitHub link: https://github.com/shyamsg/TimeSeries_MultiOmics_Review.

## Perspectives

Accounting for the temporal dimension in multi-omics studies is a rapidly expanding research theme. However, the heterogeneity of analytical approaches in current studies and the need for more systematic approaches centering around specific well-defined application tasks are clear. There is a rapidly increasing need for integrative analysis methods and open research software. Such tools are essential for supporting the practical application of the many rigorous statistical and ML methods recently introduced in this research area. Results from such studies could be expected to have an increasing impact in ecological, evolutionary, and medical research.
